# Anticarcinogenic Properties of Medium Chain Fatty Acids on Human Colorectal, Skin and Breast Cancer Cells *in Vitro*

**DOI:** 10.3390/ijms16035014

**Published:** 2015-03-05

**Authors:** Amoolya Narayanan, Sangeetha Ananda Baskaran, Mary Anne Roshni Amalaradjou, Kumar Venkitanarayanan

**Affiliations:** 1 Department of Psychology, University of Connecticut, 406 Babbidge Road, Unit 1020, Storrs, CT 06269-1020, USA; E-Mail: amoolya.narayanan@uconn.edu; 2 Department of Animal Science, University of Connecticut, 3636 Horse Barn Hill Road Ext., Unit 4040, Storrs, CT 06269, USA; E-Mails: girsan81@yahoo.com (S.A.B.); mary_anne.amalaradjou@uconn.edu (M.A.R.A.)

**Keywords:** medium chain fatty acids, anticarcinogenic properties, human colon cells, human skin cells, human breast cells

## Abstract

Colorectal cancer, breast cancer and skin cancer are commonly-reported cancer types in the U.S. Although radiation and chemotherapy are routinely used to treat cancer, they produce side effects in patients. Additionally, resistance to chemotherapeutic drugs has been noticed in cancers. Thus, there is a need for effective and safe bioprophylactics and biotherapeutics in cancer therapy. The medicinal value of goat milk has been recognized for centuries and is primarily attributed to three fatty acids, namely capric, caprylic and caproic acids. This research investigates the anticancer property of these fatty acids on human colorectal, skin and mammary gland cancer cells. The cancer cells were treated with various concentrations of fatty acids for 48 h, and cell viability was monitored by the 3-(4,5-dimethylthiazol-2-yl)-2,5-diphenyltetrazolium bromide (MTT) reduction assay. Additionally, real-time quantitative PCR (RT-qPCR) was performed to elucidate the potential anti-cancer mechanisms of the three fatty acids under investigation. Capric, caprylic and caproic acids reduced cancer cell viability by 70% to 90% (*p* < 0.05) compared to controls. RT-qPCR data indicated that these natural molecules produced anticancer effects by down-regulating cell cycle regulatory genes and up-regulating genes involved in apoptosis. Future research will validate the anticancer effect of these fatty acids in an appropriate *in vivo* model.

## 1. Introduction

Despite advances in the diagnosis and treatment of cancer, the disease remains a leading cause of mortality in most countries [1]. In the United States, cancer is the second most common cause of death and accounts for one of every four deaths [2]. It is also the leading cause of mortality in people less than 85 years of age in the U.S. [3]. Among the various cancers, colorectal cancer, breast cancer and skin cancer (non-melanoma) represent the most commonly-diagnosed forms and the leading cause of cancer-related mortality in the U.S. [2,4,5,6,7,8,9].

The commonly-employed treatment strategies against cancer include surgery, radiotherapy and chemotherapy, where the latter two are increasingly used in metastatic cancers. However, radiation and chemotherapeutic drugs produce an array of side effects in patients, including fatigue, anemia, hair loss, nausea, vomiting, diarrhea, skin problems, nerve and muscle problems, kidney and bladder irritation and sexual and fertility problems [10]. In addition, acquired tumor resistance to drugs has been noticed in some chemotherapeutic agents, thereby causing an obstacle to successful treatment [11,12]. Thus, there is a need for effective and safe biomolecules to prevent and treat cancers [8]. The ideal candidate molecule is one with minimal side effects, but efficacious at killing cancerous cells. Natural products from a variety of sources have served as a basis for the development of novel drugs, thereby contributing to human health and well-being [13,14,15]. Although the anticarcinogenic properties of several natural products have been reported, many more still remain to be discovered [16,17,18].

Since biblical times, animal products have been used for their medicinal value in various cultures [19,20]. The medicinal value of goat milk has been recorded in ancient Jewish literature [21]. Similarly, the health benefits and therapeutic value of goat milk have been recognized for centuries in Asian and Mediterranean countries [22,23,24,25]. The medicinal value of goat milk is primarily attributed to three fatty acids, namely capric (C10:0), caprylic (C8:0) and caproic (C6:0) acids, which constitute about 15% of the total fatty acid content in goat milk [25]. Capric, caprylic and caproic acids are medium chain fatty acids containing 10, 8 and 6 carbons, respectively, and are approved as generally recognizable as safe by the U.S. Food and Drug (21CFR172.860) Administration. The objective of this study was to determine the inhibitory effect of capric, caprylic and caproic acids on the growth of cultured human colon, breast and skin cancer cells. Additionally, the potential molecular mechanism(s) behind the anticancer property of the three fatty acids was investigated.

## 2. Results and Discussion

### 2.1. Results

#### 2.1.1. Medium Chain Fatty Acids Inhibit the Growth of Human Cancer Cell Types

**Figure 1:**
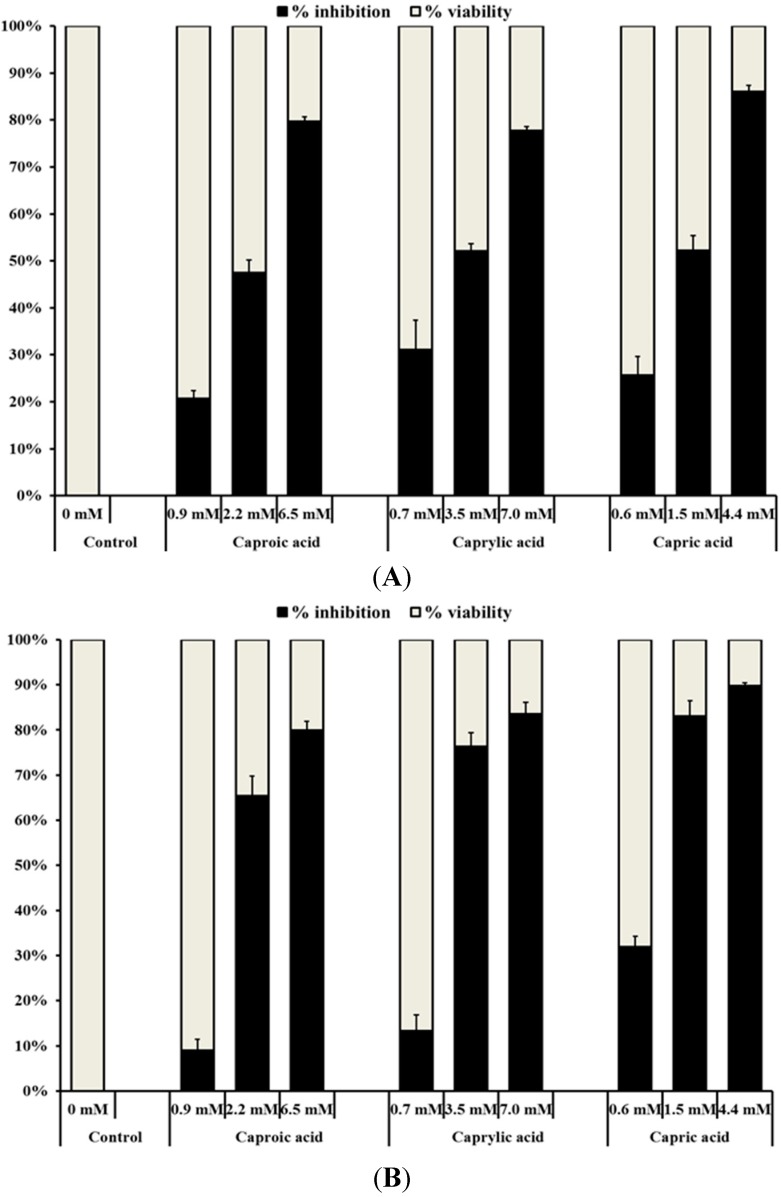

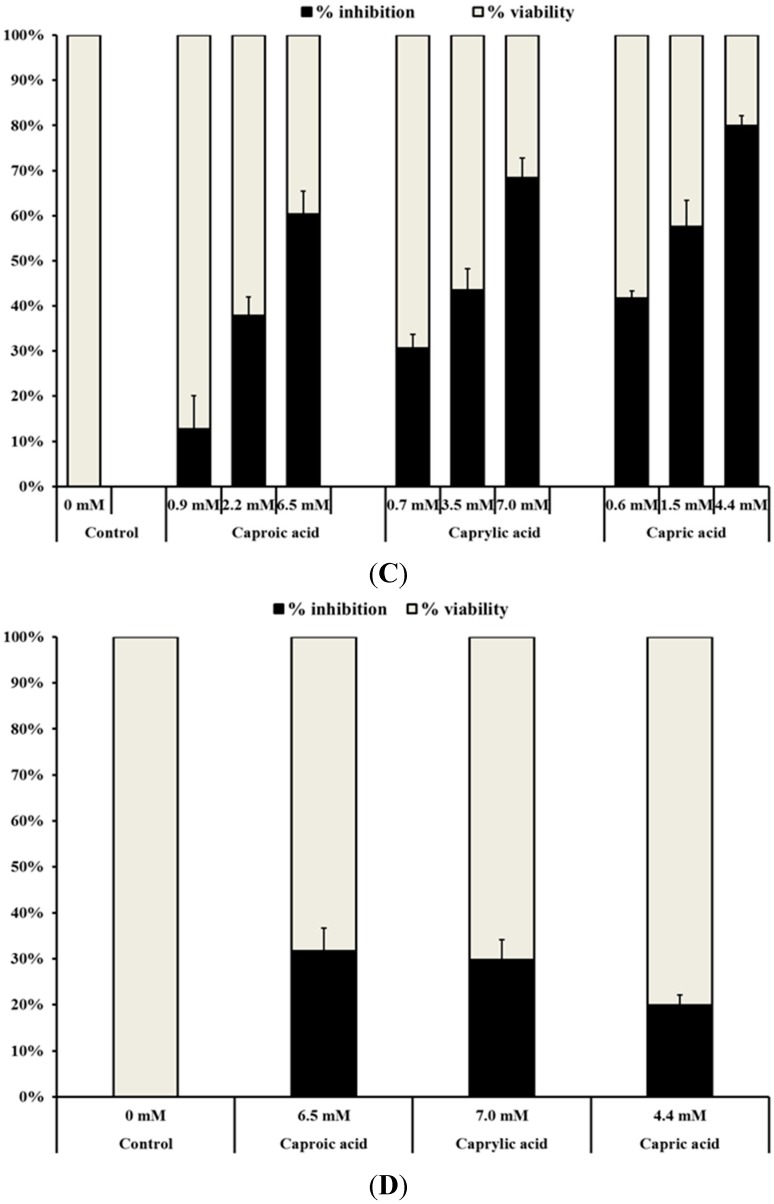
Inhibitory effect of goat milk medium chain fatty acids on the growth of (**A**) human colorectal carcinoma cells; (**B**) human skin epidermoid carcinoma cells; (**C**) human mammary gland adenocarcinoma cells and (**D**) normal human colon fibroblast cells. Cancerous cells (approximately 10^5^ cells/mL) were seeded into 96-well tissue culture plates and incubated at 37 °C for 24 h prior to treatment. After 24 h, fresh growth medium containing the various concentrations of capric (0.60, 1.50 and 4.40 mM), caprylic (0.70, 3.50 and 7.00 mM) or caproic acids (0.90, 2.20 and 6.50 mM) was added into each well. Following incubation at 37 °C for 48 h, the growth inhibitory activity of the fatty acids was investigated using the MTT colorimetric assay.

The results from cell culture assays revealed that all three goat milk fatty acids studied significantly (*p* < 0.05) inhibited the viability of the three cancer cell lines (Figure 1A–C). At the respective highest concentration, the three fatty acids reduced the viability of colon and skin cancer cells by approximately 75% to 90% compared to the control. On the other hand, the maximum growth inhibition produced by the highest fatty acid concentrations on breast cancer cells ranged from 60% (caproic acid) to 80% (capric acid). It was also observed that the inhibitory effect of capric, caprylic and caproic acids on the growth of normal colon cells was minimal compared to that on cancer cells (Figure 1D). A maximum reduction of 20%, 29% and 31% was observed in the growth of normal colon cells treated with capric, caprylic and caproic acid, respectively.

#### 2.1.2. Medium Chain Fatty Acids Modulate Critical Genes Required for Cell Progression and Apoptosis in Human Cancer Cell Types

**Figure 2:**
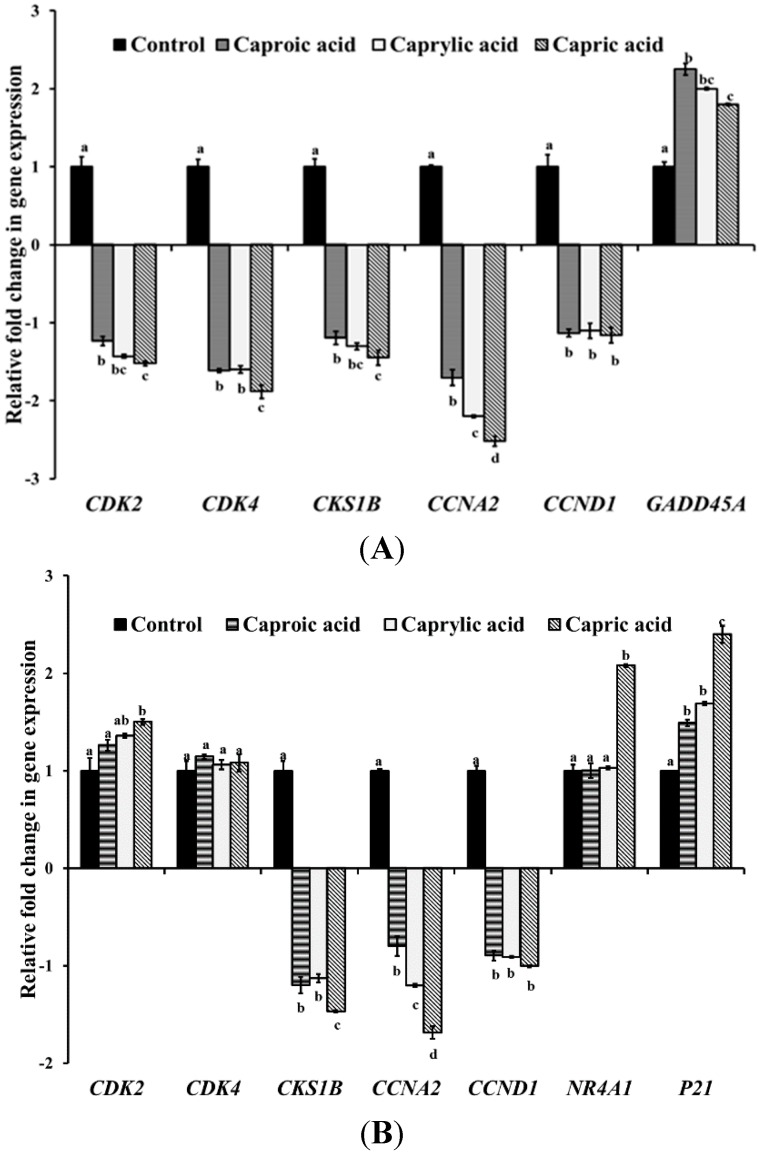

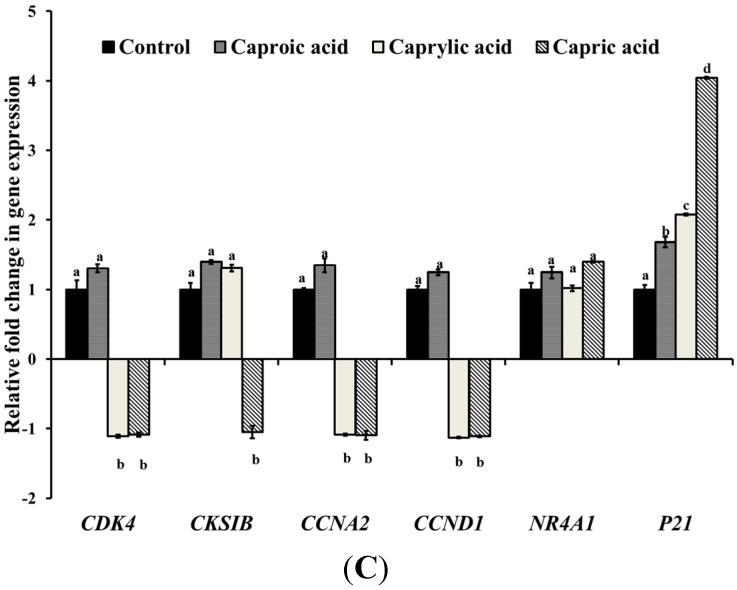
Effect of goat milk medium chain fatty acids on the expression of cell cycle progression- and apoptosis-associated genes in (**A**) human colorectal carcinoma cells; (**B**) human skin epidermoid carcinoma cells and (**C**) human mammary gland adenocarcinoma cells. Relative gene expression was assayed using the StepOne Plus Real-Time PCR System. The data were normalized to the endogenous control (16s RNA), and the level of candidate gene expression between treated and untreated samples was compared to study relative gene expression and the effect of capric, caproic and caprylic acids on candidate genes. Bars marked with letters (a, b, c, d) are significantly different at *p* < 0.05.

The RT-qPCR results from the three cancerous cell types are provided in Figure 2A–C. The three fatty acids substantially down-regulated the genes important for cell cycle division and progression in colon cancer cells, including *CDK2* (cyclin-dependent kinase 2), *CDK4* (cyclin-dependent kinase 4), *CKSIb* (CDC 28 protein kinase 1B), *CCNA2* (cyclin A2) and *CCND1* (cyclin D) genes in HCT-116 cells (Figure 2A). In unison, the fatty acids also up-regulated the Gadd45a gene, which plays a role in apoptosis in colon cancer cells. Similarly, the fatty acids down-regulated the cell division genes (*CKSIb*, *CCNA2* and *CCND1*) and up-regulated *NR4A1* (peroxiredoxin 1) and *P21* (cyclin-dependent kinase inhibitor 1) genes necessary for apoptosis in the skin cancer cells (Figure 2B). In breast cancer cells, a similar trend in the gene expression profile was observed, but the cell division and progression genes (*CDK4*, *CKSIb*, *CCNA2* and *CCND1*) were down-regulated only by capric and caprylic acids (Figure 2C). However, the expression of P21 associated with apoptosis was up-regulated by all three fatty acids.

**Figure 3:**
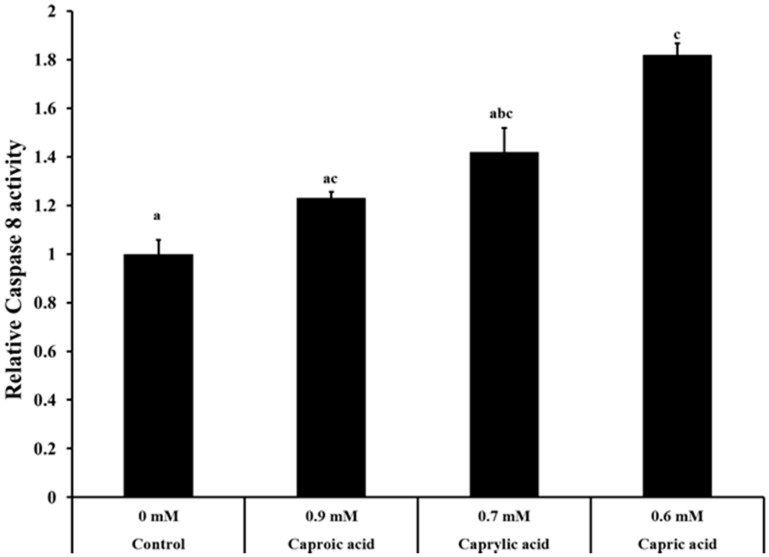
Effect of goat milk medium chain fatty acids on caspase-8 activity in human colorectal carcinoma cells. The HCT-116 cells were treated with capric (0.60 mM), caprylic (0.70 mM) or caproic acids (0.90 mM) for 24 h. After 24 h, caspase 8 activity was determined using caspase-8 fluorimetric assay. Bars marked with letters (a, b, c) are significantly different at *p* < 0.05.

#### 2.1.3. Goat Milk Medium Chain Fatty Acids Induced Activation of Caspase-8 and Apoptosis in Colon Cancer Cells

The caspase-8 fluorimetric assay is based on the hydrolysis of the peptide substrate, acetyl-Ile-Glu-Thr-Asp-7-amino-4-methyl coumarin, by caspase-8, resulting in the release of the fluorescent 7-amino-4-methylcoumarin moiety. The signal intensity is proportional to the amount of cleaved substrate, which, in turn, is dependent on caspase activity. This, in turn, is dependent on the apoptotic cell population present in treated and untreated cells. The effect of capric, caprylic and caproic acids on capsase-8 activity in colon cancer cells is provided in Figure 3. The relative caspase 8 activity of cells treated with fatty acids was higher than that of the control group. Cells treated with capric acid had a 1.8-fold increase in caspase-8 activity compared to the control (Figure 3).

### 2.2. Discussion

In recent years, the use of natural products for preventing and treating cancer has received renewed attention [4,26,27]. A few natural sources that have yielded potential anticancer compounds include plants, microorganisms and marine organisms [28]. The health benefits and the medicinal use of goat milk have been recognized in ancient Jewish literature [21] and Asian countries [22,24,25]. The medicinal properties of goat milk are attributed to capric, caprylic and caproic acids, which constitute about 15% of total fatty acid content in goat milk [25,29,30]. However, most information on the therapeutic benefits of goat milk is based on anecdotal evidence. Moreover, the anticarcinogenic potential of goat milk or its major fatty acids has not been previously determined. Therefore, this study investigated the anticancer property of capric, caprylic and caproic acids on three common cancer types in the U.S., namely colorectal, skin and breast cancers [2].

This study investigated the ability of capric, caprylic or caproic acid to inhibit the proliferation of human colorectal carcinoma, human skin epidermoid carcinoma and human mammary gland adenocarcinoma cells. The cancer cells were treated with various concentrations of fatty acids for 48 h, and their effect on cell viability was monitored. The concentrations of fatty acids used in the growth inhibition assay were selected based on preliminary experiments that were performed using a wide range of concentrations for each fatty acid. As indicated before, all three fatty acids significantly (*p* < 0.05) inhibited the cell proliferation of human colorectal carcinoma, skin epidermoid carcinoma and mammary gland adenocarcinoma cells (Figure 1A–C). The growth inhibitory effect was concentration dependent for all three fatty acids, with the highest concentration producing the greatest anticancer effect. It was also observed that capric acid exhibited the strongest inhibitory effect, especially on colon and skin cancer cells, followed by caprylic and caproic acids. Although the reason behind the differences in their efficacies is not known, the anticancer efficacy generally diminished with the decrease in the number carbon atoms present in the fatty acid; the most effective capric acid contains the highest number of carbons [10], followed by eight and six carbons in caprylic and caproic acids, respectively.

In order to elucidate the potential molecular mechanisms behind the anticancer property of the three fatty acids, the effect of capric, caprylic and caproic acids on the expression of genes critical for cell cycle and apoptosis in cancer cells was investigated. The RT-qPCR results revealed that the fatty acids down-regulated the expression of these genes, thereby supporting the results from the grown inhibition assay. The proteins encoded by genes *CDK2* (cyclin-dependent kinase 2), *CDK4* (cyclin-dependent kinase 4), *CKSIb* (CDC 28 protein kinase 1B), *CCNA2* (cyclin A2) and *CCND1* (cyclin D) have been characterized to play a role in cell division in HCT-116 cells [31,32]. These cell cycle regulatory genes are important for the multiplication and progression of cancerous cells. Real-time qPCR results from skin cancer cells also indicated that the three fatty acids decreased the expression of cell division genes (*CKSIb*, *CCNA2* and *CCND1*) (Figure 2B). Further, the expression of *NR4A1* (peroxiredoxin 1) and *P21* (cyclin-dependent kinase inhibitor 1) genes necessary for apoptosis was up-regulated by caprylic acid and all three fatty acids, respectively (Figure 2B). P21 has been reported to inhibit the activity of several cyclin-Cdk complexes, such as cyclin A-Cdk2, cyclin D1-Cdk4 and cyclin E1-Cdk2, thereby resulting in cell cycle arrest at the G1–S transition checkpoint [33,34]. These aforementioned genes are also associated with delaying and stopping the cell cycle of oncogenic cells, thus playing a critical role in apoptosis [32,35]. *Gadd45a* has also been reported to be associated with many biological processes, including apoptosis [36]. Apoptosis is the physiological process by which undesirable or unwanted cells are destroyed during development and other biological processes [4]. In fact, a variety of chemopreventive molecules have been demonstrated to induce apoptosis in premalignant and malignant cells, both *in vitro* and *in vivo* [37]. Thus, the inhibitory effect of capric, caprylic and caproic acids on the growth of colorectal, skin and breast cancer cells could potentially be attributed to their down-regulating effect on genes critical for cell cycle progression and division and up-regulation of genes associated with cell cycle arrest and apoptosis. However, there could be other mechanisms involved, and further research on the effect of these fatty acids on various pathways of cancer progression is needed.

Apoptosis is a physiological process of programmed cell death that is essential for the maintenance of homeostasis in multicellular organisms. Caspases are cysteine-requiring aspartate proteases involved in apoptosis. Caspases belong to a highly conserved family of cysteine proteases with specificity for aspartic acid residues of their substrates [38]. Caspase-8 is localized at the top of the hierarchy of the caspase cascade and is a member of the “upstream” or initiator family of caspases. Caspase-8 activates downstream caspases [3,6,7], which lead to apoptotic death of the cells [38,39]. The relative caspase-8 activity of cancer cells treated with capric, caprylic and caproic acids was significantly greater than that of the control cells not exposed to any fatty acid. These results concur with the results from the gene expression studies and underscore that one of the mechanisms by which the goat milk fatty acids inhibited the proliferation of cancer cells is by increasing apoptosis in the cells.

Although this study demonstrates the anticancerous property of caprylic acid, a recent study by Yamasaki *et al*. [40] showed that 3 mM caprylic acid failed to inhibit bladder cancer cell migration and invasion, despite reducing cell proliferation. Additionally, Jansen *et al*. [41] reported that intake of high fat or full fat dairy products that are rich in saturated fatty acids, including caprylic, capric and caproic acid, were found to increase the risk of pancreatic cancer. These observed differences in the anticancerous effect of these fatty acids could be attributed to the difference in the cancer type being studied, the difference in the concentration of the active ingredient used and other variables associated with the study. For example, Yamasaki *et al*. [40] evaluated the anticancerous property of 1 mM caprylic acid on bladder cell invasion and adhesion, whereas our current study utilized caprylic acid up to 7 mM. Additionally, the present study investigated the effect of caprylic acid on the viability of skin, colon and breast cancer cells, which are histologically different from bladder cells. The aforementioned differences in the anticarcinogenic effect of caprylic acid highlight the need for in-depth investigations to fully understand the potential application of medium chain fatty acids in cancer prevention and therapy.

## 3. Experimental Section

### 3.1. Cell Lines and Cell Culture

The cell lines, HCT-116 (human colorectal carcinoma cells), A-431 (human skin epidermoid carcinoma cells), CCD-33Co (normal human colon fibroblast) and MDA-MB-231 (human mammary gland adenocarcinoma cells), were purchased from American Type Culture Collection (ATCC, Rockville, MD, USA). These cell lines have been widely used for studying the anticancer properties of numerous molecules [4,42,43,44]. HCT-116 and A-431cells were grown in McCoy’s 5a Modified Medium and Dulbecco’s Modified Eagle’s Medium (DMEM, Life Technologies, New York, NY, USA) containing 10% fetal bovine serum (FBS, Atlanta Biologicals, Norcross, GA, USA), respectively. The MDA-MB-231 cells were cultured in DMEM containing 10% FBS and 1× non-essential amino acids (NEAA, Life Technologies, NY, USA). The CCD-33Co cells were maintained in Minimal Essential Medium (MEM, Life Technologies, NY, USA) containing 10% FBS. All cell lines were maintained at 37 °C in a humidified incubator under 5% carbon dioxide atmosphere.

### 3.2. Growth Inhibition Assay

The growth inhibitory activity of capric, caprylic and caproic acids (Sigma-Aldrich, St. Louis, MO, USA) was investigated using the MTT colorimetric assay, as described previously [45,46]. The cancerous cells were grown under the aforementioned respective growth conditions, and approximately 10^5^ cells/mL were seeded into 96-well tissue culture plates (Falcon, Franklin Lakes, NJ, USA) and incubated at 37 °C for 24 h prior to treatment. After 24 h, the spent medium was removed, and fresh growth medium containing the various concentrations of capric (0.60, 1.50 and 4.40 mM), caprylic (0.70, 3.50 and 7.00 mM) or caproic acids (0.90, 2.20 and 6.50 mM) was added into each well. Cells not treated with any fatty acid served as controls. Following incubation at 37 °C for 48 h, 50 µL of 0.5 mg/mL MTT reagent (ATCC) were added into each well and further incubated for 2 h at 37 °C. The solution containing the MTT reagent and cells was aspirated, and the purple formazan crystals formed were dissolved in 100 µL of dimethyl sulfoxide (Sigma-Aldrich). Then, the optical density of the solution was measured at 570 nm using a micro-plate reader. Appropriate blanks (without cells) were also included, and the blank reading was subtracted from the sample readings. The cell viability of fatty acid-treated samples was expressed as a percentage relative to untreated control samples. Besides the three cancer cell lines, the effect of capric, caprylic and caproic acids on normal human colon fibroblasts (CCD-33Co) was also determined. All experiments were conducted in triplicate and repeated at least three times.

### 3.3. Determination of the Anticancer Mechanisms of Goat Milk Medium Chain Fatty Acids

#### 3.3.1. RNA Isolation, cDNA Synthesis and Real-Time Quantitative RT-PCR (RT-qPCR)

The effect of capric, caprylic and caproic acids on the expression of cell cycle and apoptosis (programmed cell death) genes was investigated using real-time quantitative polymerase chain reaction (RT-qPCR) [47]. The cancer cells treated with each fatty acid separately (0.90 mM caproic acid, 0.7 mM caprylic acid and 0.60 mM capric acid) for 48 h were harvested by centrifugation (3600× *g*, 20 min, at 4 °C).The resulting cell pellet was resuspended in RNAlater solution (Applied Biosystems, Foster City, CA, USA) and stored at −20 °C until the RNA was extracted. Cancer cells not treated with the fatty acids served as controls. Total RNA from control and fatty acid-treated cancer cells (HCT-116, A431 and MDA-MB-231) was extracted using the RNeasy mini kit (Qiagen, Valencia, CA, USA), according to the manufacturer’s instructions. RNA (1 µg) from the control and treatment samples was normalized and reverse-transcribed into cDNA using the Superscript ІІ Reverse transcriptase kit (Invitrogen, Carlsbad, CA, USA). The synthesized cDNA was used as the template for RT-qPCR amplification of the target genes used in this study. Specific primers for the genes tested and the HPRT1 gene encoding hypoxanthine phosphoribosyl transferase [48] (endogenous control) were designed using Primer Express software (Applied Biosystems). The primer sequences of the target genes are provided in Table 1. The primers were synthesized by IDT DNA (Integrated DNA Technologies Inc., Coralville, IA, USA). Real-time PCR was performed using the StepOnePlus™ Real-Time PCR System (Applied Biosystems) and SYBR green technology (Life Technologies, NY, USA). The biological replicates were analyzed in duplicate, and the experiment was repeated three times. Data were normalized to the endogenous control, and the level of candidate gene expression between fatty acid-treated and untreated samples was compared to study the relative gene expression and the effect of the three fatty acids on the tested genes.

**Table 1:** Primers used in this study. Primers were designed using Primer Express 3.0 from Applied Biosystems.

Primer	Sequence (5ꞌ→3ꞌ)	NCBI Reference No.
P27F P27R	CGGTGGACCACGAAGAGTTAA GGCTCGCCTCTTCCATGTC	NM_004064.3
CDK4F CDK4R	AGGCGACTGGAGGCTTTTG GTGGCACAGACGTCCATCAG	NM_000075.2
CDK2F CDK2R	GACTCGCTGGCGCTTCA CGTGCCCTCTCCGATCTTT	NM_001798.3
CCND1F CCNDIR	CGTGGCCTCTAAGATGAAGGA CGGTGTAGATGCACAGCTTCTC	NM_053056.2
CCNA2F CCNA2R	CAACAGAGGTTGGGAGTGGAA GCATTTCTCGTCTGTTAATTTGCA	NM_001237.3
GADD45AF GADD45AR	GATGTGGCTCTGCAGATCCA ATGTCGTTCTCGCAGCAAAA	NM_001924.2
CKS1BF CKS1BR	CCACTACCCAAGAAACCAAAGAA GCTGTGTAAAGCTTGAGGCTGAA	NM_001826.2
HPRT1F ^‡^ HPRT1R ^‡^	CGTCTTGCTCGAGATGTGATG GCACACAGAGGGCTACAATGTG	NM_000194.2
NR4A1F NR4A1R	AGGGCTGCAAGGGCTTCT GGCAGATGTACTTGGCGTTTTT	NM_002135.3
P21F P21R	TGGAGACTCTCAGGGTCGAAA GCGTTTGGAGTGGTAGAAATCTG	NM_000389.3

‡ This gene is commonly used as an endogenous control to normalize gene expression in real time qPCR data [48].

#### 3.3.2. Caspase-8 Activity Assay for Apoptosis

The HCT-116 cells were treated with capric (0.60 mM), caprylic (0.70 mM) or caproic acids (0.90 mM) for 24 h. After 24 h, caspase 8 activity was determined using the Caspase-8 Assay Fluorimetric kit (Sigma-Aldrich), according to the manufacturer’s instructions. Briefly, the treated and untreated cells were washed with PBS and lysed with cell lysis buffer. Then, the suspension was centrifuged for 1 min at 10,000× *g*, and the supernatant (cytosolic extract) was used for the assay. The reaction was carried out with reaction buffer and the acetyl-Ile-Glu-Thr-Asp-7-amino-4-methyl coumarin (Ac-IETD-AMC) caspase substrate. The plate was incubated in a fluorescence plate reader (Synergy 2 Multi-Mode Reader, BioTek, Winooski, VT, USA) for 4 h, and fluorescence was read every 10 min. The excitation and emission wavelengths of fluorescent 7-amino-4-methylcoumarin (AMC) are 360 and 440 nm, respectively.

### 3.4. Statistical Analysis

For each treatment and control, data from the independent replicate trials were pooled, and the results were expressed as the means ± standard error (SE). For statistical analysis of the data, one-way ANOVA was applied using the Data Analysis ToolPak in Microsoft Office Excel 2007 (Microsoft, Farmington, CT, USA). The differences were considered significant at *p* < 0.05.

## 4. Conclusions

The results from this study revealed that capric, caprylic and caproic acids exerted significant anticancer activity on cultured human colorectal, skin and breast cancer cells and could potentially be used to prevent and/or treat these cancers. However, future research to validate the anticancer potential of capric, caprylic and caproic acids in an appropriate *in vivo* model is necessary. In addition, further studies using a whole genome approach, such as transcriptome analysis of treated and control cancer cells, would yield comprehensive information on the mechanisms of the anticancer effects of these fatty acids.
